# An Examination of Packing Methods for Grafts to Prevent Freezing Injury during Transportation for Liver Transplantation

**DOI:** 10.3390/jcm12144703

**Published:** 2023-07-15

**Authors:** Kei Kurihara, Taihei Ito, Naohiro Aida, Takashi Kenmochi, Mamoru Kusaka, Hiroto Egawa

**Affiliations:** 1 Department of Transplantation and Regenerative Medicine, School of Medicine, Fujita Health University, Toyoake 470-1192, Japann-aida@fujita-hu.ac.jp (N.A.); kenmochi@fujita-hu.ac.jp (T.K.); 2 The Japan Society for Organ Preservation and Biology, Palace Side Building, 1-1-1 Hitotsubashi, Chiyoda-ku, Tokyo 100-0003, Japan; 3 The Japan Society for Transplantation, D’s VARIE Shin-Otsuka Building 4F, 5-3-13 Otsuka, Bunkyo-ku, Tokyo 112-0012, Japan

**Keywords:** graft package, organ preservation, freezing injury, University of Wisconsin solution

## Abstract

Background: University of Wisconsin solution (UW) may freeze at temperatures below −0.7 °C, damaging the graft. The present study assessed the effectiveness of the liver graft package protocol, which recommends filling a package with sufficient liquid to prevent grafts from sustaining freezing injury. Methods: We filled ice cubes at two temperatures (−80 and −20 °C) around packages and performed a comparative study with four groups based on the temperature and filling of the second layer with lactated Ringer’s solution (LR) (A: −80 °C, LR−; B: −80 °C, LR+; C: −20 °C, LR−; D: −20 °C, LR+). The bovine liver was used as a graft and preserved for 6 h in the first isolation bag filled with UW. Results: While temperatures dropped below −0.7 °C at some points for 6 h in groups A, B, C, they never dropped to −0.7 °C in group D. The macroscopic findings in groups A, B, C showed freezing of the UW and grafts, but no such results in group D. A pathological study including electron microscopy showed freezing injury in groups A, B, and C but no significant changes in group D. Conclusions: The graft package protocol prevents freezing of the UW and liver grafts.

## 1. Introduction

In Japan, the number of liver transplants from brain-dead donors has been on the rise since the revision of the Organ Transplant Law in 2010 [1,2,3,4]. However, while 80 cases of liver transplantation from brain-dead donors are performed annually, and it has been established as a standard treatment method, many processes are required in the field of transplantation medical care, from organ transportation to transplantation surgery and the procured organ must be maintained at some degree of cold ischemia [5,6]. Therefore, low-temperature packaging technologies, such as appropriate temperature control and organ packing, are indispensable in transplant medicine.

To maintain a low temperature, ice cubes are usually packed around the procured organs and stored in a portable container, such as a cool box, for transportation. These ice cubes are stored in a freezer or cold storage after being made with an ice machine set at ≤−18 °C to prevent the growth of bacteria by both the Japanese Industrial Standard (JIS) [7] and the International Electrotechnical Commission (IEC) [8]. When using this protocol, the cryogenic ice at −18 °C used to fill the portable containers might contact the preservation solution, such as the University of Wisconsin solution (UW). Therefore, it must be kept in mind that the UW filled around the procured organ may freeze at a temperature below the freezing point, with the organ potentially suffering freezing damage. Indeed, experimental verification performed by Matsumoto et al. showed that the freezing point of UW was −0.7 °C [9]. Potanos et al. [10] reported that UW used to store a liver graft for pediatric transplantation was exposed to low temperatures below the freezing point during transportation. This resulted in primary nonfunction (PNF) after transplantation due to cryoinjury. As a result of post facto verification in this case report, the following causes were assumed: (1) the amount of UW in the first bag packed with hepatic graft was only 400 mL, which is less than the amount recommended by the United Network for Organ Sharing (UNOS) [11]; and (2) the second layer was filled with solid ice cubes instead of a liquid, such as saline or slush ice. A few cases of freezing injury of the procured liver have also been reported in Japan, so the possibility of freezing injury during transporting of organs intended for transplantation must be carefully considered.

In response, the Japan Society for Transplantation (JST) 7 established a standard protocol for organ packaging (Table 1). 

The present study verified whether or not the Japanese standard protocol for organ packaging can prevent freezing injury during organ transportation.

## 2. Materials and Methods

### 2.1. Graft Liver Packaging Model

**Figure 1:**
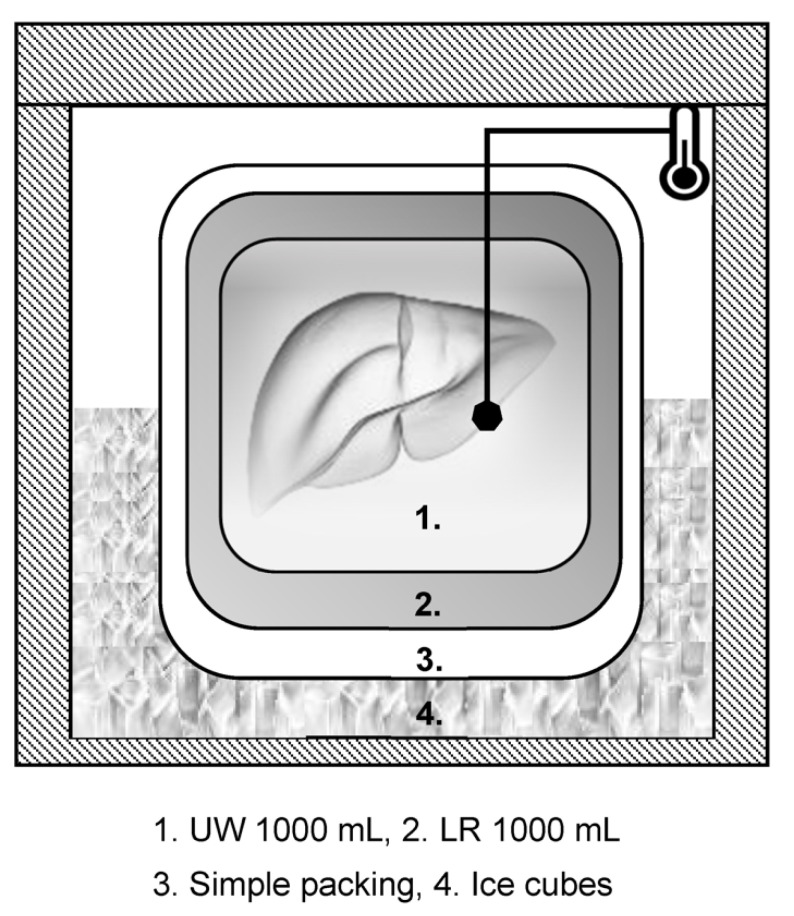
**Schematic diagram of the experimental liver graft.** (1) In the first layer, approximately 1 kg of bovine liver as an experimental model was soaked in 1000 mL of University of Wisconsin solution (UW). (2) The second layer was filled with 1000 mL of lactated Ringer’s solution. (3) The third layer was packed without liquid and placed in the cooler box. (4) The cooler box was filled with ice cubes at −80 °C or −20 °C. A thermometer was installed to monitor the temperature of the UW in the first layer.

A schematic diagram of the experimental liver graft based on the standard protocol for organ packaging presented by the JST is shown (Figure 1).

In this study, commercially available beef livers from animals slaughtered by the Nagoya Meat Public Corporation on the day of the experiment were preserved at about 6 °C before the experiment as a liver graft model. Since the weight of the human liver is approximately 1000 g, the bovine livers were trimmed to a weight of 1000 g as a liver graft model and used in the experiment. To prevent tissue disintegration during manipulation that would affect the experimental results, the left lobe of the liver was cut on one or two sides to make one block.

The first isolation bag (3M Steridrape^®^ 50 × 50 cm, 3M Corporation, Saint Paul, MN, USA) was packed containing the liver graft model with 1000 mL of UW (Belzer UW Cold Preservation Solution^®^; Astellas Pharma Inc., Tokyo, Japan) and then packed with more two bags as well. After initial wrapping, the second layer was filled with 1000 mL of lactated Ringer’s solution (LR; Sollacto S^®^; Terumo Corporation, Tokyo, Japan), and the third layer was simply closed without any liquid.

### 2.2. Low-Temperature Environment for Graft Liver Preservation

This experiment used a cooler box for liver graft preservation with an internal volume of 20 L (specimen transportation box manufactured by Sekisui Material Solutions Co., Ltd., Tokyo, Japan) as a specimen box for organ transportation. Ice cubes with a side length of about 2 cm made by an ice cube machine (IM-230AM-1; Hoshizaki Co., Ltd., Aichi, Japan) were used to fill the cooler box. To bring the ice cubes to temperatures of −20 and −80 °C, ice cubes for filling were placed in a deep freezer (MDF-U33V-PJ; Panasonic Co., Ltd., Tokyo, Japan) for ≥24 h at −20 and −80 °C, respectively, and to create a low-temperature environment, the cooler box was filled with about 15 L of these ice cubes.

### 2.3. Temperature Monitoring

Regarding temperature monitoring, a data logger for temperature measurement (TR-71nw; T&D, Nagano, Japan) was used to monitor the temperature of the UW in the first layer, including the experimental graft liver. The measurement probe terminals were fixed to the surface of the experimental graft liver and the inner wall of the cooler box, and the temperature was measured continuously.

### 2.4. Histopathological Examinations

After cold storage of the experimental graft liver in a cooler box for 6 h, the macroscopic findings of both the UW and the experimental graft liver were observed. A 1-cm-square piece of liver tissue was then excised and collected as a specimen for a histopathological examination. The liver surface capsule was sectioned and microscopically examined, focusing on the central vein as a guide to tissue structure. For hematoxylin and eosin (HE) staining, all specimens were fixed with a 10% buffered formalin solution, entrusted to KAC Co., Ltd. (Kyoto, Japan) embedded in paraffin, and observed at a magnification of 400×.

Electron microscopy was outsourced to Hanaichi Electron Microscopy Laboratory (Aichi, Japan). All specimens were prefixed with 2% glutaraldehyde in 0.1 M cacodylate buffer at 4 °C, dehydrated in graded ethanol solutions, briefly washed with propylene oxide, and embedded in epoxy resin. Conventional ultrathin sections were cut and double-stained with uranyl acetate and lead citrate for 5 min. These ultra-thin sections were examined with an electron microscope (H-600; Hitachi, Tokyo, Japan) at 5000× magnification.

### 2.5. Experimental Design

- **Experiment 1**

To confirm that the cooler box used in the experiment was effective at maintaining a constant temperature, as a model that deviated from the standard packaging, we used a packaging model that did not use LR in the second layer and filled it with −80 °C ice for 24 h while monitoring the temperature of the experimental liver graft and in the box. After 24 h, the macroscopic appearance of the UW and experimental liver graft was observed.

- **Experiment 2**

**Figure 2:**
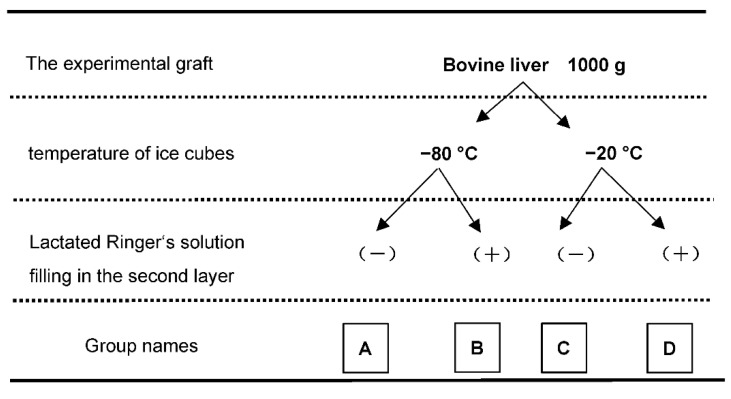
**Experimental design.** Four experimental groups were established, A: ice cubes at −80 °C, lactated Ringer’s solution (LR) (−), B: −80 °C, LR (+), C: −20 °C, LR (−), D: −20 °C, LR (+).

We set 2 temperature conditions: at −80 °C assuming a cryogenic environment and at −20 °C assuming ice that could be used in normal clinical practice for the experiments. (Figure 2)

Comparable experiments were performed at these temperatures while filling the second layer with or without LR. As shown in the figure, the following groups were established: A, at −80 °C and LR−; B, at −80 °C and LR+; C, at −20 °C and LR−; and D, at −20 °C and LR+. Continuous temperature monitoring for six hours from the start of the package and sample collection after cold storage were repeated three times. In the overall experiment, six commercial bovine livers were purchased and trimmed, resulting in 12 packaging models.

## 3. Results

### 3.1. Experiment 1

**Figure 3:**
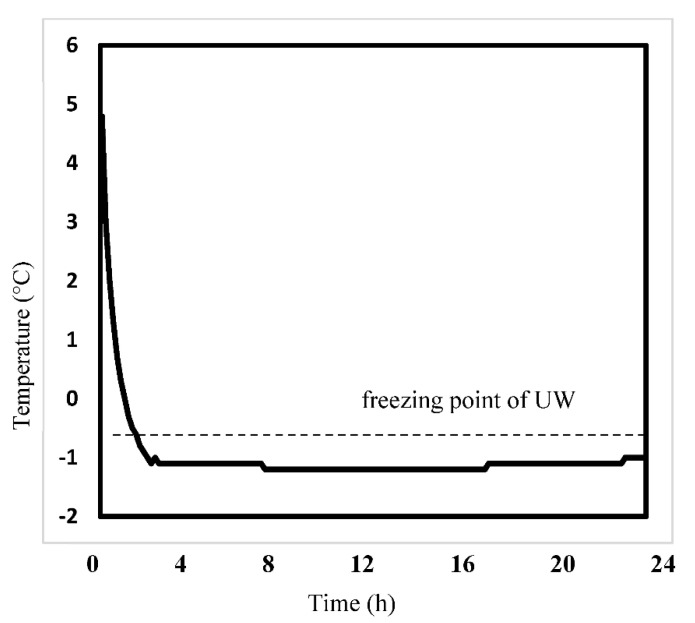
**Temperature changes in the University of Wisconsin solution over 24 h (Experiment 1).** The experimental graft was placed in a cooler box filled with ice cubes at −80 °C for 24 h, and the temperature change in the University of Wisconsin solution (UW) was monitored. The temperature of the UW dropped below the freezing point (−0.7 °C) after about 20 min and plateaued at −1.2 °C after about 2 h, remaining there for 24 h.

The temperature in the cooler box was measured by filling it with ice cubes at −80 °C. About 20 min after the start of cold storage, the temperature of the UW with the experimental liver graft dropped below freezing (<−0.7 °C). Two hours after the start of cooling, the temperature converged to −1.2 °C, and the temperature remained almost constant until 24 h after the end of the observation (Figure 3). 

After 24 h, the cooler box was opened, and a macroscopic observation showed that the UW and experimental liver graft were completely frozen.

### 3.2. Experiment 2

Given the results of UW temperature monitoring, which showed a plateau at −1.2 °C at 2 h after the start of cooling, and the change in the appearance of the experimental liver graft in Experiment 1, and considering the time required for liver transportation in Japan, Experiment 2 was performed with a 6-h cold storage model for comparison among groups. 

Table 2 shows the results of UW temperature change monitoring during cold storage with the experimental graft. In all four groups, the temperature immediately decreased from the start of monitoring and stabilized after one to three hours. In groups A and B, which involved filling the box with ice cubes at −80 °C, the temperature fell below −0.7 °C in 2 out of 3 experiments, regardless of LR presence. These changes occurred after approximately two to three hours.

In groups C and D, which involved filling the box with ice cubes at −20° C, the UW was ≤−0.7 °C during the measurement period in 2 out of 3 experiments in group C (without LR filling). However, in group D (with LR filling, the recommended standard protocol for organ packaging), the temperature never dropped to −0.7 °C for 6 h. 

**Figure 4:**
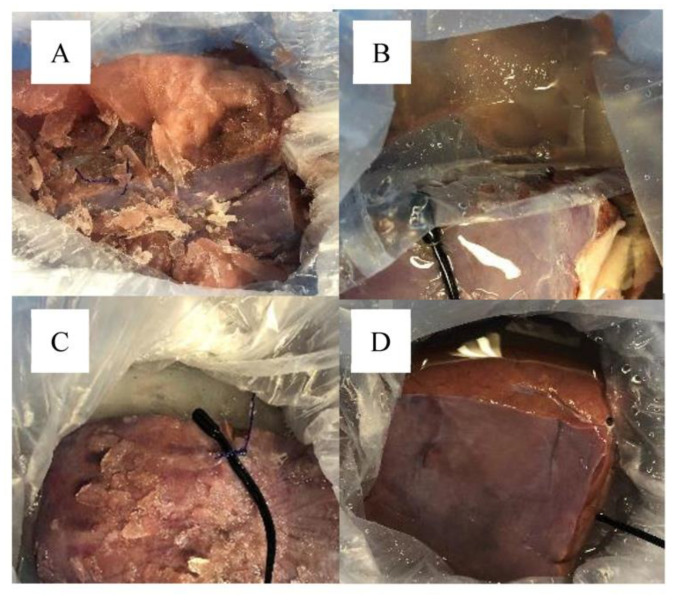
**Macroscopic findings of the experimental graft after six hours of cold storage.** Six hours after cold storage, the package was opened, and the macroscopic results of the University of Wisconsin solution (UW) and the experimental liver graft were observed. The UW and experimental graft were frozen in groups A, B, and C, whereas no freezing was observed on the UW or experimental graft in group D, filled with ice cubes at −20 °C with lactated Ringer’s solution in the second layer.

In addition, in groups A, B, and C, which reached below-freezing temperatures, the macroscopic findings showed freezing of the UW and the experimental graft (Figure 4), 

In group D, which was filled with LR in the second layer, neither the UW nor the experimental graft showed any freezing macroscopically.

**Figure 5:**
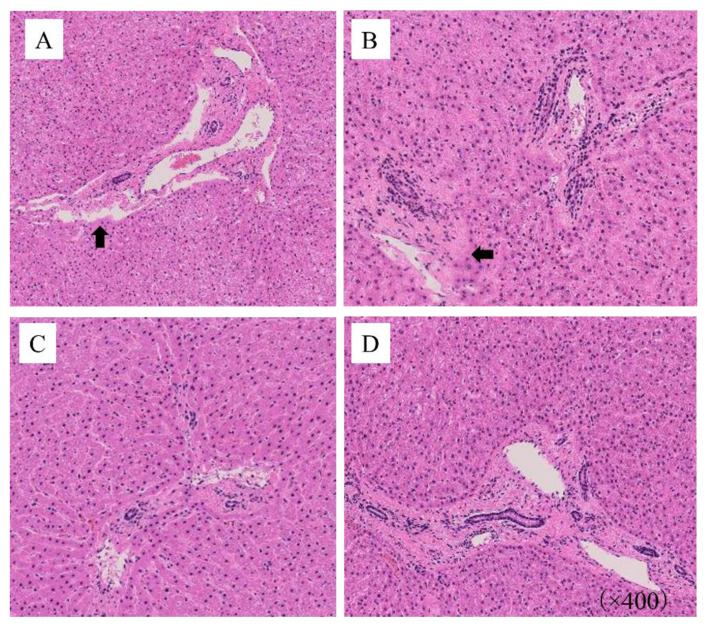
Microscopic findings with hematoxylin and eosin (HE) staining of the experimental graft after six hours of cold storage. Microscopic results with HE staining showed the expansion of sinusoids in the areas (black arrows), the disordered architecture of the pericentral vein tissue, and vacuolar degeneration in the cytoplasm in groups A and B with ice cubes at −80 °C. In contrast, no remarkable findings were noted in groups C or D, with no microscopically marked difference between these two groups.

Figure 5 shows representative microscopic findings with HE staining. At 400× magnification, regardless of the presence of LR in the second layer, the arrangement between hepatocytes in groups A and B filled with ice cubes at −80 °C changed irregularly, and these findings were particularly remarkable around the central vein. In addition, diffuse changes in vacuolar degeneration were observed in the hepatocyte parenchyma. 

In contrast, the normal hepatocyte arrangement was maintained in groups C and D filled with ice cubes at −20 °C under observation at 400× with HE staining, and no significant differences were observed in the tissue structure.

**Figure 6:**
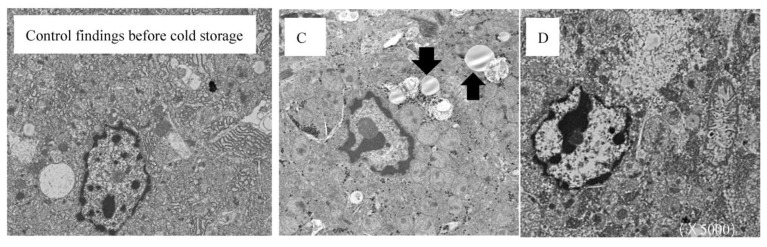
**A comparison of electron microscopy findings of the experimental grafts with ice cubes at −20 °C.** The electron microscopy findings of the experimental liver graft were compared with those before cold storage as the control. The circularization of intracellular organelles and crystallization of water were observed in the area (black arrow) in group C, which lacked lactated Ringer’s solution (LR) in the second layer. There was no significant change in the electron microscopy findings in group D, which had LR in the second layer, compared to the control.

Additional observations with an electron microscope were performed to confirm the impact of LR filling in the second layer on structural changes in the hepatocytes. In group C (LR−), the membrane structure of the intracellular organelles was significantly disturbed, and crystallized ice chips appeared in the cells in the areas shown in the figure, indicating freezing injury. In contrast, in group D (LR+), the same observations as in the control before cold storage were obtained concerning the structure of the nucleus and the membranes in the intracellular organelles (Figure 6)

## 4. Discussion

To perform liver transplantation safely, graft preservation to prevent PNF from organ harvesting and resumption of blood flow in the recipient is one of the essential factors. The first report of organ preservation with normothermic storage was made by Carrel et al. [12] in the 1930s, followed by Statzl et al. [13], who reported the cold storage method for grafts in the 1960s. Since then, cold storage has been the major preservation method for grafts.

However, following a report of a liver transplantation case in which freezing injury caused by excessive low-temperature exposure during transportation resulted in PNF, it became necessary to review the conventional cold preservation packaging method. Potanos et al. [10], who reported the first case of freezing injury of graft liver during transportation, pointed out the small amount of liver preservation solution in the first layer compared to the recommendation by UNOS and the direct attachment of the ice cubes in the second layer to the first layer, which included the UW and graft liver, as potential reasons for the sustained injury. Transplant physicians should always keep in mind that the freezing point of UW is −0.7 °C, and ice cubes obtained from the ice machine may be below that temperature, so direct contact would cause the UW to freeze.

There is no standard protocol for liver packaging in Japan, with approaches varying among regions, facilities, and transplantation teams. However, given our experiences with graft freezing, a standard protocol for liver packaging is needed to prevent such issues going forward. The JST developed a standard protocol for liver packaging based on these experiences, and the present study was conducted to verify whether the protocol was appropriate. The first experiment to verify the cooling status of a cooler box used for normal liver transport showed that the temperature in the cooler box filled with ice cubes at −80 °C, to mimic packaging, reached a plateau at −1.2 °C after about 2 h and remained at this temperature for 24 h. These findings clarified that the commercial-based cooler box used for liver transportation reaches a temperature below the freezing point of the UW when filled with supercooled ice cubes and maintains a sufficiently low-temperature environment for 24 h.

Given this finding, we decided to set a cold storage time of 6 h in the next experiment, assuming the typical transport time for such cases in Japan. We also assessed the effectiveness of filling the second layer with LR as temperature interference. As shown in Table 2, the monitored temperature showed some variation. However, in group D, the temperature never dropped below −0.7 °C, and no freezing was observed during any tests. Thus, filling the second layer with LR, as is recommended in the standard protocol for liver packaging by the JST, avoids direct touching between the UW and ice cubes and thus prevents grafts from being exposed to an excessively low temperature.

The pathological findings in groups A and B, which involved filling with ice cubes at −80 °C, revealed the disturbance and rupture of the tissue architecture around the central vein, findings that were similar to a report of hepatocellular freeze injury in rats [14]. However, no marked differences in findings were noted between groups C and D on examining HE-stained high-magnification images. Therefore, an additional examination with electron microscopy was performed, which revealed that while the structure in the hepatocytes was maintained in group D, there was apparent denaturing of the hepatocyte structure in group C. The low-temperature damage observed on electron microscopy has also been previously reported [15], showing findings compatible with our own.

Tullius et al. [16] reported that the accumulation of intravascular and interstitial crystals and the formation of extracellular and intracellular ice in the organ tissue were the main causes of freezing injury in organs and were particularly harmful to the vascular system. These crystals resulted in endothelial and hepatocyte damage and caused PNF after the transplantation of cryo-damaged organs. To prevent such cryo-damage, a previous report [17] showed that the cell structure could be preserved without crystallizing the intracellular water with ultra-rapid freezing. However, while this is theoretically possible, our current understanding is too poor to apply it effectively in clinical practice.

A pathological study including electron microscopic findings in the present study revealed that cell degeneration induced by low temperatures could be prevented by applying the standard protocol for organ packaging, involving filling the second layer with LR in an environment with ice cubes at −20 °C, which is considered possible for liver transportation. However, when ice cubes are used at −80 °C, regarded as an extreme condition and not a clinical situation, graft freezing cannot be prevented, even when the second layer is filled with LR.

A limitation and weakness of this experiment is that the temperature measurement was limited to a single point on the liver surface per specimen, and the results were limited to a restricted range. The uniformity of the temperature distribution in the liver during cooling cannot be ruled out. Therefore, as a prospect, more accurate results could be obtained if measurements could be taken at multiple points. Also, as a point to add, how the packing method affects graft function and outcome after transplantation is a very important question. We believe the validity of the standard packing method confirmed in this study must be confirmed in a living transplant model rather than organ units.

## 5. Conclusions

Depending on the freezer, the temperature of the ice cubes filled in the cooler box used for graft transportation may be −18 °C, as specified by JIS and IEC standards, instead of 0 °C, at which point livers can freeze. The present findings showed that, as long as the second layer is filled with enough liquid as temperature interference, avoiding direct contact between the UW and the ice cubes helps prevent excessive low temperatures and freezing of the UW and grafts. It was found that safe liver packaging is possible in the current method of operation without any additional effort or new resources. Thus, the effectiveness of the standard protocol for liver packaging recommended by the JST was confirmed, and all efforts should be made to comply with this protocol.

## Figures and Tables

**Table 1:** The standard protocol for organ packages was established by The Japan Society for Transplantation.

	First Layer		Second Layer		Third Layer	
Transplant Graft	Liquid	Ice	Liquid	Ice	Liquid	Ice
	(Type/Quantity)		(Type/Quantity)		(Type/Quantity)	
Heart	Preservation solution 500–1000 mL	none	saline or LR/500–1000 mL	none	saline or LR/500–1000 mL	none
Lung	Preservative solution/appropriate amount	none	Facility Judgment	Facility Judgment	Facility Judgment	Facility Judgment
Liver	UW/1000 mL	none	saline or LR/1000 mL	none	none	none
Pancreas	UW/≥500 mL	none	saline or LR/≥500 mL	none	none	none
Kidney	UW is advisable, but EC is also acceptable ≥500 mL	none	Isolation bag or plastic container saline or LR/≥500 mL	Liquid only or slush ice	none	none
Small intestine	UW/1000 mL	none	saline or LR/1000 mL	none	none	none

EC, Euro Collins solution; LR, Lactated Ringer’s solution; UW, University of Wisconsin solution.

**Table 2:** Temperature changes in the University of Wisconsin solution in the experimental package.

Temperature of Ice Cubes	LR Filled in the Second Layer	Groups	Hours from the Initial Packaging						
			0	1	2	3	4	5	6
−80 °C	−	A1	12.3	−0.8	−1.1	−1.2	−1.2	−1.2	−1.2
		A2	9.4	2.7	0.1	−1.2	−1	−0.6	−0.7
		A3	16.3	4.9	1.8	0.2	0	0	0.2
	+	B1	12	0.6	−0.5	−1.1	−1	−1	−1
		B2	10.3	1.2	0.6	0.2	0.1	0	−0.1
		B3	15.1	0.7	−0.4	−1.1	−1.3	−1.4	−1.4
−20 °C	−	C1	12.9	3.1	1.1	0.3	0.1	−0.1	−0.1
		C2	13.1	1.4	−0.4	−0.9	−1	−0.9	−0.8
		C3	11	−0.4	−1.1	−0.9	−0.5	−0.4	−0.3
	+	D1	15.7	4.4	2.8	1.9	1.4	1	0.9
		D2	10.4	0.4	−0.2	−0.5	−0.5	−0.4	−0.3
		D3	9.7	1.3	0.8	0.6	0.6	0.5	0.5

LR, Lactated Ringer’s solution. Background color marked data that reached below the freezing point of the UW.

## Data Availability

The data presented in this study are available on request from the corresponding author.
